# Smart Hydrogels for Advanced Drug Delivery Systems

**DOI:** 10.3390/ijms23073665

**Published:** 2022-03-27

**Authors:** Aydin Bordbar-Khiabani, Michael Gasik

**Affiliations:** Department of Chemical and Metallurgical Engineering, School of Chemical Engineering, Aalto University Foundation, 02150 Espoo, Finland; michael.gasik@aalto.fi

**Keywords:** smart hydrogels, stimuli-responsive hydrogels, drug delivery, controlled release, drug-loaded hydrogels, biomedicine

## Abstract

Since the last few decades, the development of smart hydrogels, which can respond to stimuli and adapt their responses based on external cues from their environments, has become a thriving research frontier in the biomedical engineering field. Nowadays, drug delivery systems have received great attention and smart hydrogels can be potentially used in these systems due to their high stability, physicochemical properties, and biocompatibility. Smart hydrogels can change their hydrophilicity, swelling ability, physical properties, and molecules permeability, influenced by external stimuli such as pH, temperature, electrical and magnetic fields, light, and the biomolecules’ concentration, thus resulting in the controlled release of the loaded drugs. Herein, this review encompasses the latest investigations in the field of stimuli-responsive drug-loaded hydrogels and our contribution to this matter.

## 1. Introduction

Hydrogels, as an important class of biomaterials, can be defined as coherent systems composed of a three-dimensional polymer network, containing a huge amount of aqueous phases that cannot dissolve the network through physical and chemical interactions due to the presence of interconnections, called crosslinks [1,2,3]. Nowadays, hydrogels are an appealing type of targeted drug delivery systems and have been used in many branches of medicine and biomedical engineering, including cartilage and wound regeneration, bone tissue engineering, biosensors, electronic and soft robotic component, and inflammation relief [4,5,6].

**Figure 1:**
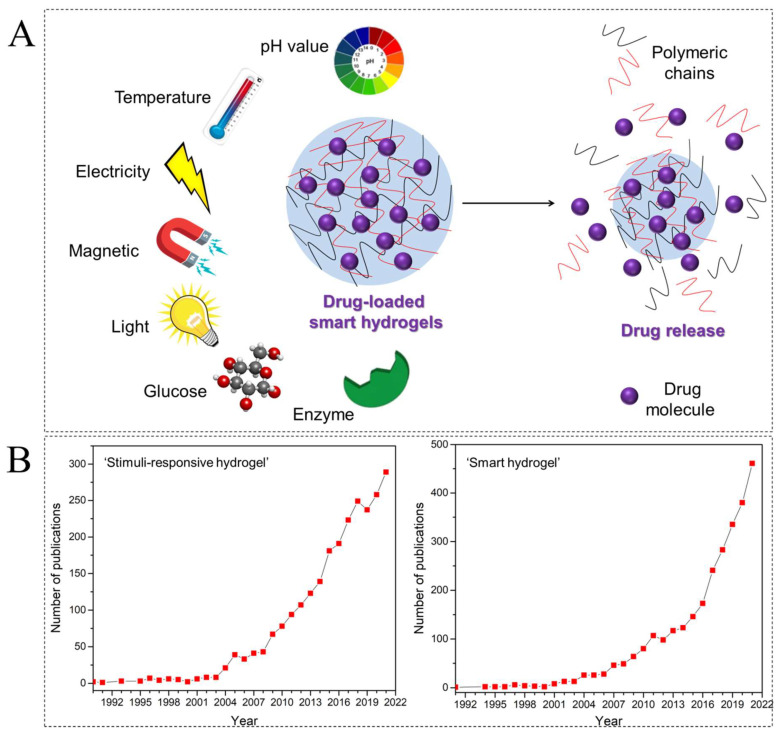
(**A**) Various external stimuli, including pH, temperature, electricity, magnetics, light, and biomolecules (including glucose and enzyme), are controlling the drug release from a smart hydrogel. (**B**) Stimuli-responsive hydrogel- and smart hydrogel-related original literature over the years. Data from Scopus, December 2021 [10].

In recent years, growing attention towards personalized pharmacotherapy and precision medicine has prompted the innovation of smart biomaterials [7,8]. Stimuli-responsive hydrogels can be regarded as smart biomaterials, and external triggers, such as pH, temperature, electrical and magnetic fields, light, and the concentration of the biomolecules, can be used to evoke drug release (Figure 1A) [9]. The ‘stimuli-responsive hydrogel’ and ‘smart hydrogel’ keywords appeared in the literature for first time in 1990 and 1991, respectively. In 2021, more than 2800 (70% of them in last 5 years) papers that are directly related with the synthesis and application of smart hydrogels have been published, as seen in Figure 1B [10].

Smart hydrogels undergo abrupt changes in their physical properties and macroscopic alterations in response to a small external trigger [11,12]. The uniqueness of these hydrogels resides in their nonlinear feedback [12]. Indeed, they can respond to triggers with a reversible, intensity-scalable, reproducible, and predictable phase volume transition and have the ability to return to their original shape after the trigger is removed [11,13]. These transitions include changes in the physical state, solvent interactions, shape and solubility, conductivity, and hydrophilicity [14].

Using smart hydrogels in drug delivery systems can reduce the dosing frequency, maintain the desired therapeutic concentration in a single dose, and minimize the drugs’ side effects by preventing the accumulation of the drugs in non-target tissues [15,16]. Moreover, smart hydrogels have an easy preparation process and are an ideal option for prolonged-release systems with incorporated drugs [17,18]. In this review, we do not intend to provide an exhaustive synopsis of the field of hydrogels—which is vast—but highlight advances and curiosities in the previous five years about stimuli-responsive hydrogels, with selected triggers for smart drug delivery applications. The classification of stimuli-responsive hydrogels along with their key features, properties, and applications are enlisted in Table 1.

## 2. pH-Responsive Hydrogels (PRHs)

**Figure 2:**
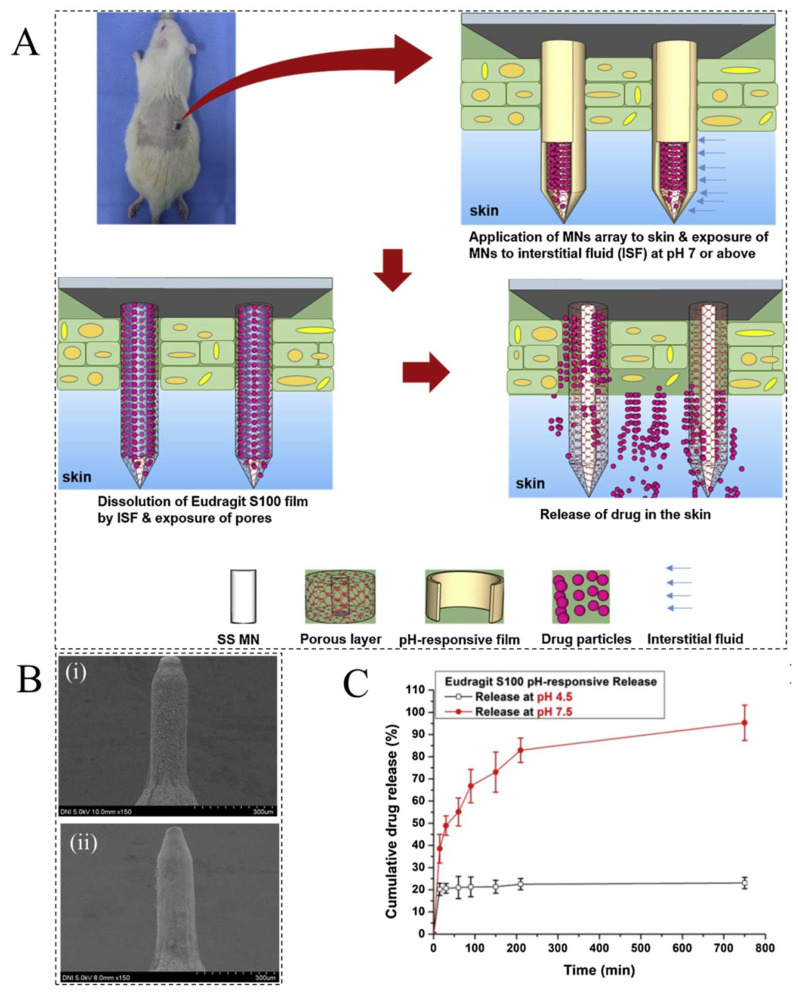
(**A**) Schematic illustration of a wound pH-dependent release system based on hydrogel-coated MNs; scanning electron microscopy (SEM) images of (**B(i)**) MN with a porous PLGA coating (**B(ii)**) MN with both porous PLGA and Eudragit S100 coatings; and (**C**) drug release profile for MNs cultivated in the wound pH (pH 7.4) and healthy skin pH (pH 4.5) media. Reprinted with permission from [43].

PRHs are high molecular polymers that undergo a phase or volume transition when the pH value of the external medium changes [37]. PRHs are usually developed by the polyelectrolytes that contain weak acidic or basic function groups. Therefore, they can be classified into two major categories, namely, cationic and anionic hydrogels, which have alkaline groups (such as –NH_2_) and acidic groups (such as –COOH) on their molecular chains, respectively [38,39]. The swelling of PRHs is affected by the pH value of the surrounding medium at the pKa and pKb values of the pendant acidic and basic groups [38]. pH variations directly affect the interactions between solvent molecules and polymer chains through the following mechanisms [19]. In anionic PRHs, when the pH of the biological environment exceeds the pKa value of the acidic groups in the polymer chain, due to the ionization of the acidic groups, negative and positive charges are formed on the polymer chain and in the solution, respectively [40]. In contrast, when the pH value of the environment is less than the pKb value of the alkaline group in cationic PRHs, the basic groups will be ionized (protonated), resulting in more negative and positive charges on the polymer chain [41]. As a result, in both PRHs, the hydrophilicity of the polymer chains and the electrostatic repulsion between the chains are enhanced, causing the polymer network to swell. pH variations can occur in the body due to certain diseases such as chronic wounds, inflammation, cancer, and tumors, which are used for targeted drug delivery to specific organs and tissues [42]. Recently, drug-loaded PLGA/Eudragit S100 coatings were deposited on stainless steel microneedles (MNs) for smart release of encapsulated therapeutics in response to wound pH levels to enhance the wound-healing process (Figure 2A,B) [43]. Eudragit S100 is an anionic PRH based on methyl methacrylate and methacrylic acid. The presence of –COOH groups in the polymer chain causes the pH-responsive behavior. For a healthy skin pH (acidic microenvironments), the pendant groups are uncharged whilst at pH values greater than their pKa (pKa~4) (i.e., above pH 7 (wound pH)), the polymer chains begin to disentangle and release the encapsulated drug. As shown in Figure 2C, 76% of the loaded drug was released in wound pH conditions during the first incubation hour.

After implantation of the biomedical devices, immune cells play a crucial role in the whole osteointegration process, including the chronic inflammatory response [44]. During inflammatory conditions immune cells and osteoclasts release reactive oxygen species (ROS) and chlorine-based acids, which significantly drop the pH in the implantation site [45,46]. The pH changes at the inflammatory medium resulted in drug release from the PRHs [47,48]. Chauhan et al. developed PRHs through crosslinking oxidized pullulan with poly(ethylene glycol) (PEG) [49]. The anti-inflammatory dexamethasone (DEX) drug was loaded into the synthesized hydrogel. The hydrogels provided a pH-sensitive sustained release of DEX with 74.54 and 55.15% at pH 6.5 and 7.4, respectively. The hydrogels were also exhibited high cell viability and osteogenic activities, which make them a good candidate for bone repair applications in chronic inflammatory conditions.

**Figure 3:**
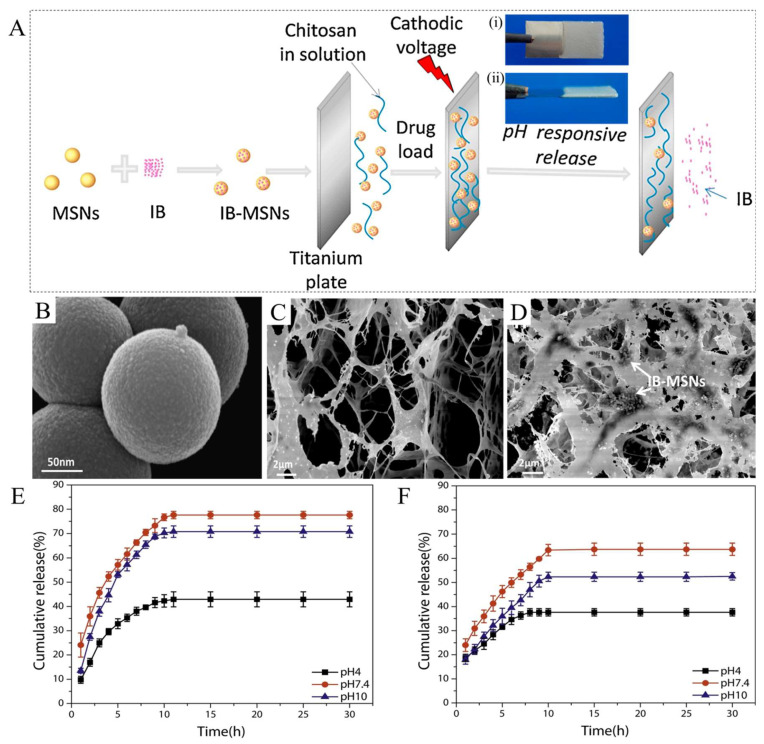
(**A**) Schematic illustration of the co-electrodeposition of the chitosan- and IB-loaded MSNs on a Ti substrate and the pH-responsive release: (**A(i)**) the front view, and (**A(ii)**) the side view of chitosan-IB-MSNs on the Ti plate; (**B**) SEM image of the MSNs; (**C**) SEM image of the chitosan; and (**D**) SEM image of the chitosan-IB-MSNs. Cumulative release profiles of IB: (**E**) IB-MSNs, and (**F**) chitosan/IB-MSNs in different pH values. Reprinted with permission from [50].

In another study, the pH-responsive system was developed on titanium (Ti) implants to release the anti-inflammatory ibuprofen (IB) drug [50]. First, IB-loaded mesoporous silica nanoparticles (MSNs) were synthesized by immersion of particles into the drug solution. Then, chitosan hydrogel and IB-MSNs were deposited on the Ti surface by the co-electrodeposition technique (Figure 3A). The SEM micrographs of the MSNs, chitosan, and chitosan-IB-MSNs are shown in Figure 3B–D, respectively. The results of the drug release studies confirmed that the release level of the hydrogel from the Ti surface is affected by the pH of the medium. As seen in Figure 3E,F, the release rate at pH 10 and pH 7.4 was faster than that of pH 4.0 in both coated samples. The release mechanism was expressed via a two-stage process: release of IB from MSNs to chitosan, and then release of IB from chitosan to the incubation medium. We expect that in the next few years, the anti-inflammatory drug-loaded PRH layers will be applied on the different metallic implants surfaces to reduce the chronic inflammation in the acidic microenvironment around the implant site.

## 3. Temperature-Responsive Hydrogels (TRHs)

**Figure 4:**
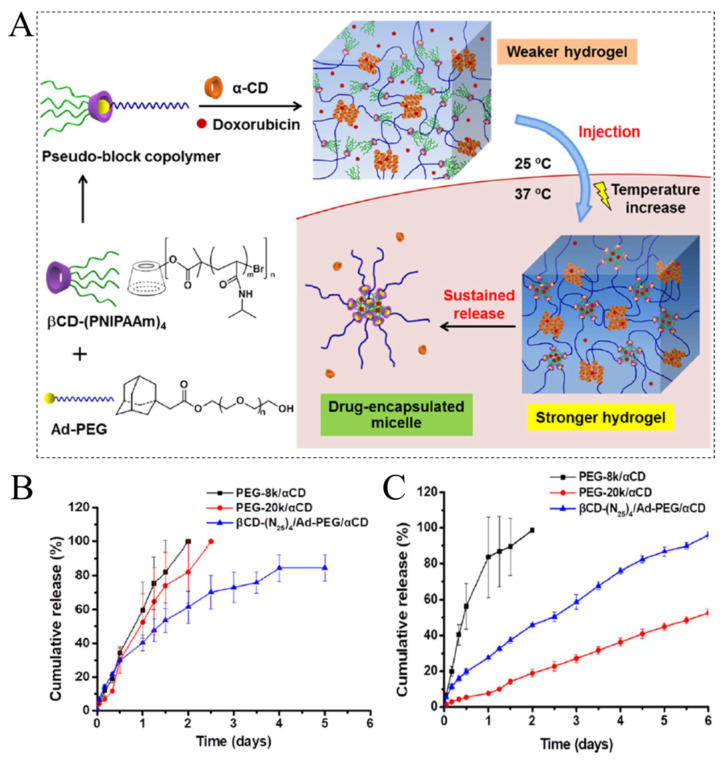
(**A**) Schematic illustration of the supramolecular hydrogel formed between the βCD PNIPAAm/Ad-PEG pseudo-block copolymer and α-CD, with a host–guest complexation between the β-CD units and adamantyl groups and the polypseudorotaxane formation between the α-CD and PEG chains. By increasing the temperature from the room temperature to body temperature, the hydrogel can release the anticancer drug. Cumulative release profiles of DOX from the synthesized hydrogels (**B**) at 37 °C and (**C**) at 25 °C. Reprinted with permission from [56].

TRHs can change their shape, size, and volume in response to physiological temperature changes and have hydrophobic groups, such as propyl, ethyl, and methyl groups on their chains [51]. The primarily used TRHs are liquid or semi-solid at ambient temperature, suffering a sol-to-gel transition when exposed to body temperature [52]. This characteristic allows a therapeutic compound to be loaded onto the hydrogel in a liquid state, which can then be easily administered and solidified upon application [21]. TRHs can be divided into lower critical solution temperature (LCST) and upper critical solution temperature (UCST) hydrogels, which exhibit non-linear responses to temperature, and upon heating of them, the solubility of the LCST and UCST hydrogels decreases and increases, respectively [21,22]. As shown on the LCST above, a higher level of insolubility and hydrophobicity can result in gel formation, whereas the LCST below indicates components of a mixture are completely soluble/miscible for all compositions [53]. Therefore, most TRHs for drug delivery systems are produced at LCST, because the phase-transition temperature of UCST is less than 25 °C, which limits their biomedical applications [54]. Polymers used to synthesis LCST-based hydrogels are poly(N,N-diethyl acrylamide) (PDEAM), poly (N-isopropylacrylamide) (PNIPAM), poly(methylvinylether) (PMVE), copolymer blocks of poly(ethylene oxide), poly(N-vinylcaprolactam) (PVC), and poly(pentapeptide) of elastin [54]. PNIPAAm is a non-biodegradable polymer showing LCST phase transition at about 32 °C in an aqueous solution, and in recent years, a lot of research has been done on it [55]. For example, a novel thermoresponsive β-cyclodextrin (β-CD)-modified PNIPAm star polymer and adamantyl-terminated poly(ethylene glycol) (Ad-PEG, in 8 k and 20 k grade) as self-assembly pseudo-block copolymers were synthesized via the host–guest interaction [56]. Afterward, with the addition of α-cyclodextrin (α-CD) into the system, a polypseudorotaxane-based supramolecular between the α-CD and PEG chain was prepared. When the temperature increased from 25 to 37 °C, the hydrogels became stronger due to the hydrophobic chains’ interactions in PNIPAM, as a dominant force. Then, the enhanced hydrogel is released, with supramolecular micelles as anticancer doxorubicin (DOX) drug carriers (Figure 4A). Figure 4B,C show the DOX release profiles from the hydrogels at 37 and 25 °C, respectively. In both temperatures, loaded-DOX could be released sustainably from hybrid hydrogels for a longer time because of the dual-stage crosslinking in their chains. As seen in Figure 3B, βCD-(N25)4/Ad-PEG/αCD hydrogels were able to release DOX for up to four days. It has been also reported that, the PEG-20 k/αCD hydrogel released DOX slower at 25 °C due to the lower solubility of CD in 25 °C water than in water at 37 °C (Figure 4C).

**Figure 5:**
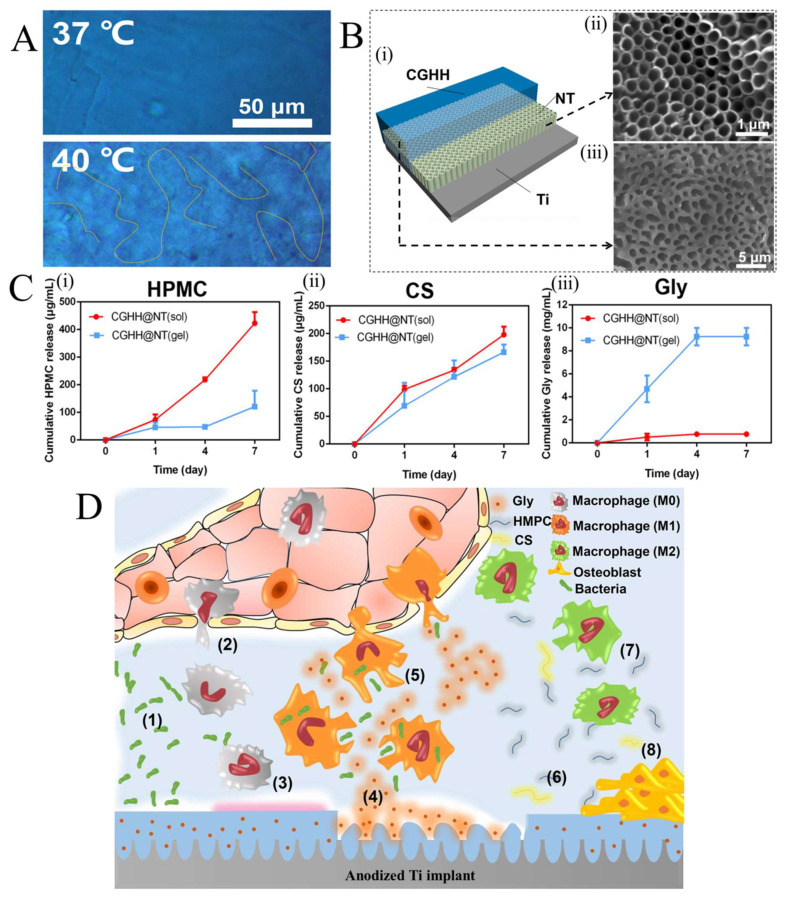
(**A**) Optical microscopic images of the hydrogel at 37 °C (sol state) and 40 °C (gel state); (**B(i)**) schematic illustration of the CGHH@NT sample; (**B(ii)**) SEM image of the NT sample; (**B(iii)**) SEM image of the CGHH@NT sample; (**C(i)**) HPMC release profile; (**C(ii)**) CS release profile; (**C(iii)**) Gly release profile; and (**D**) schematic illustration of the thermo-sensitive immunoregulation of the CGHH@NT sample. Reprinted with permission from [68].

Nowadays, Ti and its alloy are extensively used in orthopedic and dental implants for their feasible mechanical features, satisfactory corrosion resistance, and good biocompatibility [57,58,59]. However, implant-associated infection is one of the major causes of Ti and its alloys failure in the human body [60,61]. Infections will be worse if the Ti is implanted in the patient with diabetes and aging [62,63]. Most of these infections are caused by bacteria biofilms that form on biomaterial surfaces [64,65]. The surface functionalization of the implants with antibacterial and therapeutic agents offers a solution for the prevention of biofilm formation and overcoming slow bone regeneration and healing [66,67]. The drug-loaded TRHs can be also used as coating materials in bone tissue engineering for reducing the bacteria-associated infection. Recently, a TRH composed of hydroxypropyl methylcellulose (HMPC), chitosan (CS), and glycerin (Gly) was synthesized and denoted as CGHH [68]. Optical microscope photographs of the hydrogel in Figure 5A shows that CGHH was a smooth film without any cracks at 37 °C. It turned to a porous network structure at 40 °C. In the next step, the nanotubes (NT) were constructed on Ti by anodization process. Finally, the CGHH hydrogel was deposited on the anodized Ti surface by dip coating to regulate the ratio of M1 and M2 in a thermo-sensitive way, called CGHH@NT (Figure 5B). As seen in Figure 5C, the dissolution rate of HPMC, CS, and Gly in CGHH under the sol and gel state showed that, under the gel state, more Gly, and under sol state, more HPMC and CS were released from the CGHH layer. The thermo-related immunoregulatory mechanism of CGHH@NT is shown in Figure 5D schematically and the explanation of each stage along with the corresponding number is given below. During bacterial invasion of the implant’s surface (1), the organism’s innate immunity will activate, which then triggers the release of inflammatory cells, including macrophages M0, M1, and M2 (2). When the temperature exceeds the LCST of the CGHH, the hydrogel starts to transform from a sol state to a gel state and releases a lot of Gly molecules (Figure 4D(iii)) (3 and 4). In this state, macrophages polarize toward the M1 phenotype and promote inflammation, resulting in the recruitment of inflammatory cells to the infection site and the improvement of their ability to kill bacteria (5). The inflammatory response and local temperature will decrease once the bacteria have been completely killed and the hydrogel will return to the sol state (6). As seen in Figure 4D(i,ii), the hydrogel coating can release a large amount of HPMC and CS molecules under the transition temperature, which results in macrophages polarization toward the M2 phenotype (7), accelerating tissue healing and osteoblast differentiation (8). Therefore, a smart transformation between the anti-inflammatory and pro-inflammatory microenvironments will be obtained by using TRHs.

Studies in the field of bone tissue engineering have recently showed that the incorporation of bone morphogenetic protein (BMP) as a growth factor into the hydrogels can promote in vivo bone formation on the implants’ surface [69]. Li et al. constructed a porous Ti alloy (Ti-6Al-4V) and injected BMP-laden chitosan TRHs into pores of the Ti6Al4V scaffolds [70]. Rheological studies showed that the values of the storage modulus (G′) and loss modulus (G″) for the BMP-loaded hydrogel were low at the low temperature and increased with the temperature. They concluded this scaffold design provided a controlled release of BMP, thus enhancing the biocompatibility and osteogenic properties. The research trend shows that future studies on smart TRHs will focus on their drug delivery application for the treatment of chronic diseases, such as osteomyelitis, hypercholesterolemia, and diabetes.

## 4. Electrically and Magnetically Responsive Hydrogels (E and MRHs)

**Figure 6:**
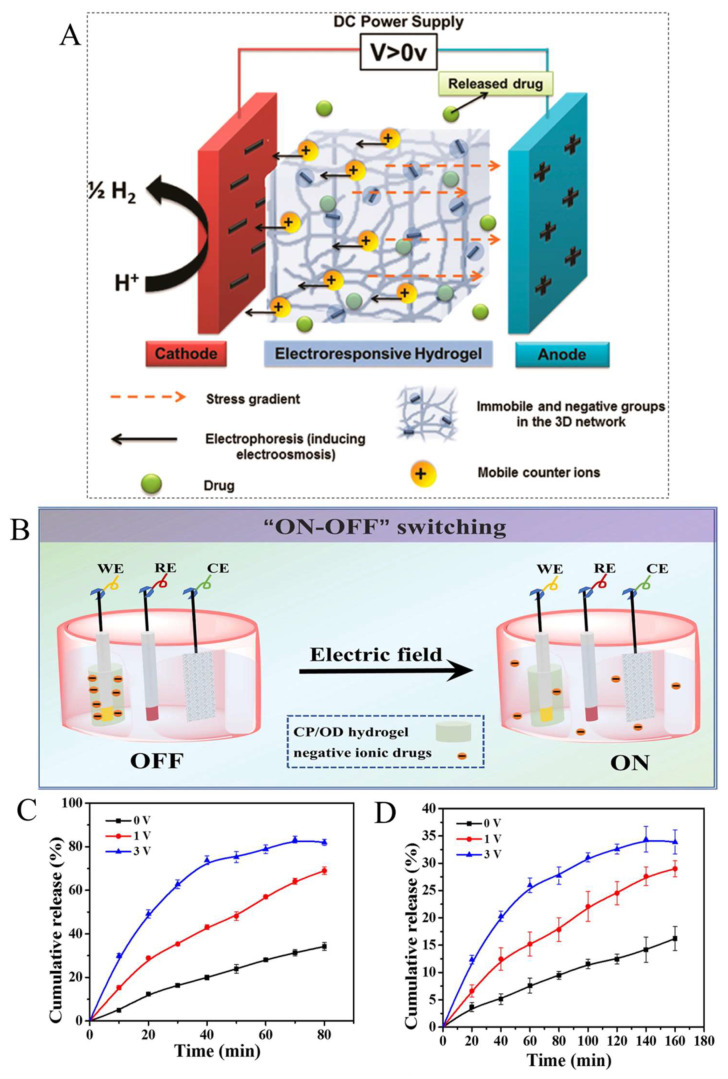
(**A**) Schematic illustration of the mechanisms for electro-induced hydrogel swelling for drug delivery applications. Reprinted with permission from [71]. (**B**) Schematic illustration of pulse release of the drug model from a CP/OD conductive hydrogel in a 3-electrode electrochemical system. (**C**) Drug release study of amoxicillin in PBS with pH 7.4 under different electric potentials. (**D**) Drug release study of ibuprofen in PBS with a pH 7.4 under different electric potentials. Reprinted with permission from [74].

ERHs refer to group of electroactive and highly hydrated hydrogels that swell or de-swell in response to an electrical current [24]. Polyaniline, polypyrrole, sulfonated styrene, polythiophene, and polyvinyl alcohol are some examples of synthetic ERHs while natural examples include chitosan, alginate, and hyaluronic acid [25]. Drug release behavior from ERHs under electrical stimulus can be controlled by three competing forces—polymer–polymer affinity, ionic pressure, and rubber elasticity—collectively calledmosmotic pressure. Disruption of the balance of these forces leads to swelling and de-swelling in ERHs [71,72]. The osmotic pressure of a hydrogel is equal to the surrounding aqueous environment at an equilibrium state. When an electrical field is applied across an ERH in an aqueous medium, H^+^ and OH^−^ ions on the polymer chains move toward different sites with opposite charges resulting in a non-uniform ion distribution, as seen in Figure 6A [71]. Therefore, the osmotic pressure is increased inside the polymer, which results in the volume transition of ERHs [73]. Indeed, the osmotic pressure difference between the hydrogel and aqueous solution is the driving force for drug release from ERHs [71,73]. Qu et al. explored the amoxicillin and ibuprofen release from hydrogels prepared by mixing a chitosan-graft-polyaniline (CP) copolymer and oxidized dextran (OD) as a cross-linker [74]. Figure 6B shows the setup consisting of a working electrode (glassy carbon coated with solidified ERHs), an Ag/AgCl reference electrode, and a platinum-mesh counter electrode schematically, which was used for in vitro drug release in a phosphate-buffered saline solution. As shown in Figure 6C,D, the cumulative release of both drugs significantly increased with the increase in the applied voltage. Approximately 82% of the amoxicillin was released from the ERH in 60 min when a 3 V potential was applied, compared to a 34% release without any electrical trigger (Figure 6C). In comparison, the ERH showed an almost 35% release of ibuprofen in 140 min when a 3 V electrical potential was applied, compared to a 15% release without any stimulation (Figure 6D).

Among the smart hydrogels, MRHs have attracted intensive researches in drug delivery, hyperthermia therapy, tissue engineering, magnetic resonance imaging, and soft actuators, owing to their unique features, including non-invasive remote actuation, quick magnetic response, and temporal and spatial control [75]. Most of the MRHs are fabricated by combining TRHs with superparamagnetic iron oxide nanoparticles (SPIONs) [27,75]. Under the effect of a magnetic field, SPIONs are vibrated, which leads to a magnetic hyperthermia, activation of the TRHs, and a change in their swelling state, thereby modulating the drug release rate [76]. Chen et al. synthesized the SPIONs-incorporated poly(N-isopropyl acrylamide) as MRHs for the controlled release of the anti-cancer drug [77]. According to Fick’s law, they reported that when the magnetic field is cut off, the hydrogel undergoes a volume transition, and the loaded drug is released into the surrounding aqueous solution. Zhang et al. [76] developed an injectable and biodegradable MRH with temperature-dependent dissolution and gelation properties for combination cancer therapy. The solution containing poly(organophosphazene), SPIONs, and the drug could transform a hydrogel at body temperature (37 °C), and then the hydrogel gradually dissolved at a temperature of 43 °C under a high-frequency alternating magnetic field (13.3 kA·m^−1^ and 366 kHz, 60 min) to enhance the drug release. Although the design of the MRHs can bring many advantages, there are still some challenges in the cytotoxicity of SPIONs through oxidative stress. Surely, the upcoming studies will reveal complex and novel behavior arising from the ability of hydrogels to decrease ROS generation via antioxidants incorporation into their structures.

## 5. Light-Responsive Hydrogels (LRHs)

**Figure 7:**
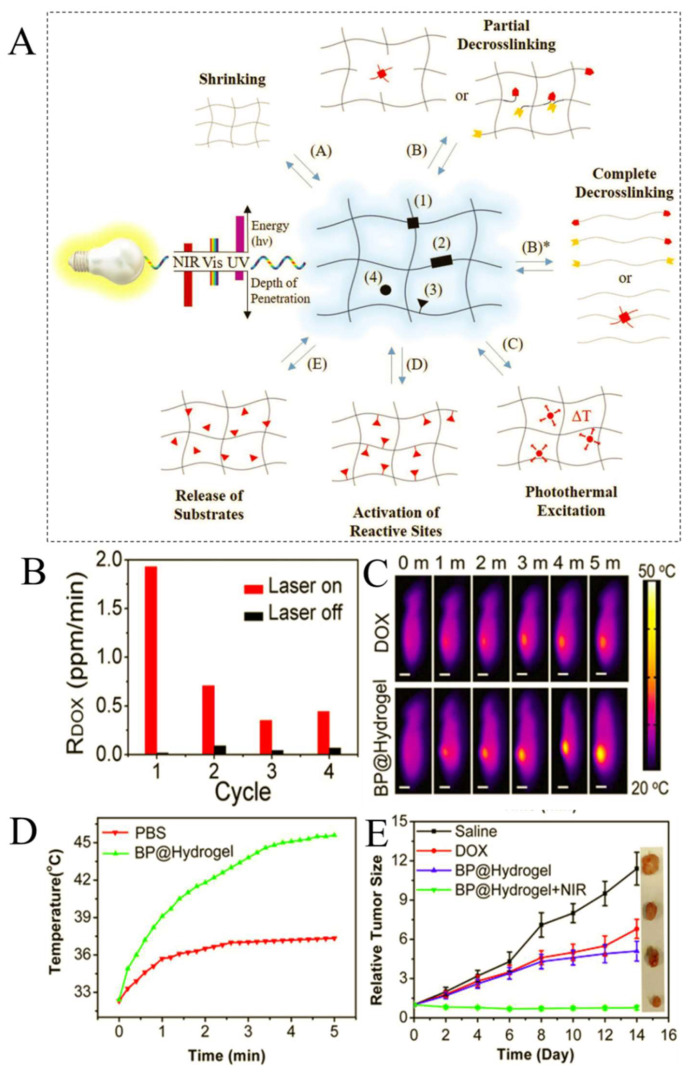
(**A**) Schematic illustration of the molecular architecture and responses of a LRH: (**A(A)**) photoresponses include shrinking, (**A(B)**) de-crosslinking partially which can be accompanied by an increase in water uptake and, consequently, an increase in hydrogel volume. (A(B*)) de-crosslinking completely leads to degradation of hydrogels. (**A(C)**) A localized increase in temperature is referred to as photothermal excitation. (**A(D)**) activation or deactivation of reactive sites, (**A(E)**) release or capture of substrates Reprinted with permission from [33]; (**B**) release rate of DOX with and without laser exposure; (**C**) thermal images of mice bearing tumors after injection of DOX or BP@Hydrogel, followed by exposure to 808-nm laser irradiation; (**D**) tumor temperature changes of mice bearing MDA-MB-231 tumors during laser irradiation as indicated in (**C**); (**E**) the corresponding growth curves of tumors in different groups of mice treated with PBS solution, DOX, BP@Hydrogel depot only, and BP@Hydrogel depot with laser irradiation. Reprinted with permission from [81].

Light as an external stimulus can promote drug release from hydrogels and offer a number of advantages such as low-cost, ease of tunability of intensity and wavelength, and a wide range of chemistries available to design the LRHs [30,31]. LRHs generally consist of specific chemical moieties, called chromophores, as the functional part of the polymer chain, and are sensitive to near-infrared radiation (NIR), visible light, and UV [30,32]. As shown in Figure 7A, the chromophores can be located in the (1) crosslinking points, (2) along the polymer backbone, (3) along the side chains, or (4) dissolved in the aqueous matrix of hydrogels [33]. Depending on the location and chemical characteristics of the particles, the response to irradiated light can be one or a combination of the following: (A) shrinking; (B) the water uptake and an increase in hydrogel volume via partial de-crosslinking; (B*) hydrogel degradation via de-crosslinking; (C) local increase in temperature through photo-thermal excitation; (D) activation or deactivation of functional groups; and (E) capture or release of the hydrogel matrix. Amongst the different wavelengths of the photochemical spectrum, NIR-responsive hydrogels have superior potentials for pharmacological treatments due to their deeper penetration in tissues (ca. 2 mm through the skin) and harmlessness [78,79].

In photodynamic therapy, as a promising method in antibacterial material design, photosensitizers are driven to create ROS after exposure to different wavelengths of light. Recently, He et al. synthesized catechol motif-modified methacrylated gelatin containing photosensitizer Chlorin e6-loaded mesoporous polydopamine nanoparticles [80]. This smart LRH was deposited on the Ti implant surface by dip coatings. Because of the ROS-generation property of Chlorin e6 under 660 nm laser stimuli, the hydrogel exhibited a significant and prompt antibacterial activity against *Escherichia coli* and *Staphylococcus aureus* bacteria when the laser power was 1 W·cm^−2^. In vitro and in vivo studies also showed that the developed smart hydrogel coating possessed fibroblast activation under the laser power of 100 mW·cm^−2^, promoting the wound repair.

An original design was proposed by Qiu et al., who developed DOX-loaded, hydrogel-encapsulated black phosphorus nanosheets (BPNSs) by using low-melting-point agarose and PEGylated BPNSs for cancer therapy [81]. The photo response of the prepared hydrogel was evaluated under an 808-nm NIR laser with a power density of 1.0 W⋅cm^−2^. Evidence exhibited the concentration of released DOX increases dramatically in the PBS solution under NIR, compared with the unchanged concentration without NIR (Figure 7B). Thermal camera analyses after in vivo injection also showed that the synthesized hydrogels had a more significant temperature rise and localized drug distribution around the tumor site than a free drug injection (Figure 7C,D). Moreover, the size of the LRH-treated tumor was significantly smaller than the other treatments, as shown in the tumor growth curve (Figure 7E). Although studies like this have been dedicated to the design and in vivo characterization of LRHs, most of the current systems are restricted to proof-of-concept studies due to the complexity of the photo-responsive materials. Nonetheless, we believe that LRHs are promising approaches to the future of local cancer therapy, especially in the case of non-surgically resectable tumors.

## 6. Biomolecule-Responsive Hydrogels

**Figure 8:**
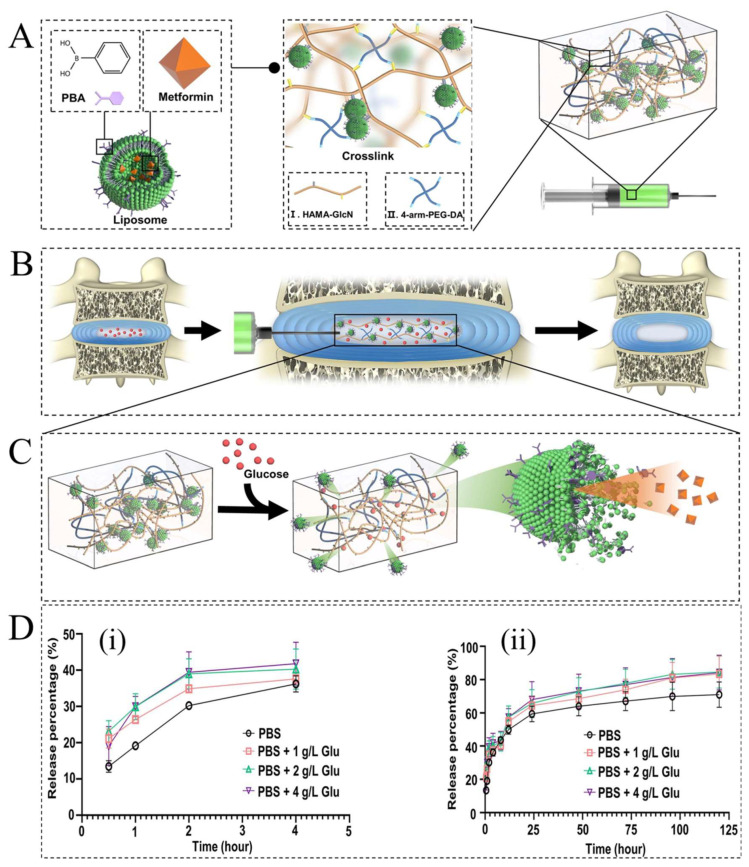
(**A**) Schematic illustration of the architecture of Met@HA-PEG as a glucose-responsive therapeutic system for regeneration of IVDD in diabetic rats. (**B**) The Met@HA-PEG was injected into the intervertebral space to construct an anti-inflammatory and antioxidant microenvironment. (**C**) The release of metformin in a high-glucose microenvironment. (**D(i)**) Metformin release kinetics of Met@HA-PEG in the first 4 h. (**D(ii)**) Metformin release kinetics of Met@HA-PEG in the first 120 h. Reprinted with permission from [89].

Biomolecule-responsive hydrogels are generally distinguished by their response to glucose, specific enzymes, protein, and the antibody molecules [34,82]. The development of materials for the self-monitoring of blood glucose to regulate the glucose level of diabetic patients is one of the hot topics in materials science and today’s medicine [82]. The glucose-responsive hydrogels (GRHs) have attracted great attention in the field of drug delivery to overcome diabetes-induced chronic inflammation [82,83]. In recent years, GRHs have been used to build an automated insulin delivery system responding to blood glucose concentrations [84]. The primary release mechanism from GRHs involves the diffusion of glucose into the membrane, where glucose is converted into gluconic acid, which lowers the pH and results in swelling in the hydrogel, followed by insulin secretion [85]. Examples of GRHs are glucose oxidase-loaded, lectin-loaded hydrogels, and hydrogels with phenylboronic acid moieties. However, these smart hydrogels can also be used to send therapeutic to tissues such as bone that have been damaged by diabetes [86]. Diabetes as a metabolic disease cause a pathological high-glucose microenvironment in tissues, which would significantly accelerate the progress of the preexist inflammation and prevent local tissue regeneration [87]. Diabetic patients with bone tissue damages, such as osteoporosis fractures and intervertebral disc degeneration (IVDD), struggle with chronic inflammatory disease too, which is difficult and costly to treat and control in clinical practice [88]. Most recently, a GRH based on hyaluronic acid (HA) and polyethylene glycol (PEG) loaded with metformin (Met@HA-PEG) was designed for injection to intervertebral disc spaces and introduced to create a stable anti-inflammatory microenvironment via dynamically adjusted to the change of glucose concentration in IVDD (Figure 8A–C) [89]. As seen in Figure 8D,E, in vitro biomechanical analyses on the intervertebral discs of rats proved that injection of the Met@HA-PEG provided strong mechanical support for degenerated intervertebral discs due to its elasticity. The drug release assessment of Met@HA-PEG showed that 84.23% and 70.96% of the total metformin were released in the PBS solutions with 4 g·L^−1^ and 0 g·L^−1^ glucose, respectively (Figure 8D), indicating the sensitivity of the hydrogel to changes in glucose. Moreover, the synthesized GRH could decrease the ROS effects on mitochondria and increase the generation of extracellular matrices in the nucleus pulposus cells.

Living cells undergoes all major changes as a result of enzymes [63]. Enzymes are biomolecules that play an indispensable role in many biological and chemical reactions within cells [90]. They can act as a natural trigger and be used to design enzyme-responsive hydrogels (EZRHs) [90,91]. One of the most popular methods for the synthesis of EZRHs is the incorporation of an enzyme-catalyzed reaction [91]. To develop this type of hydrogel, the following points should be considered. They must contain enzyme-identifying elements, such as linkers. The linkers in the hydrogel structure must be readily available to the enzymes for identification. Moreover, the reaction between the linker and the enzyme must cause physical and chemical changes and then degradation or morphological transformation of the EZRHs [92,93]. In drug-loaded EZRHs, therapeutic molecules can be dispersed in the hydrogel structure by encapsulation and they are released locally based on enzymatic activity and hydrogel degradation [94].

**Figure 9:**
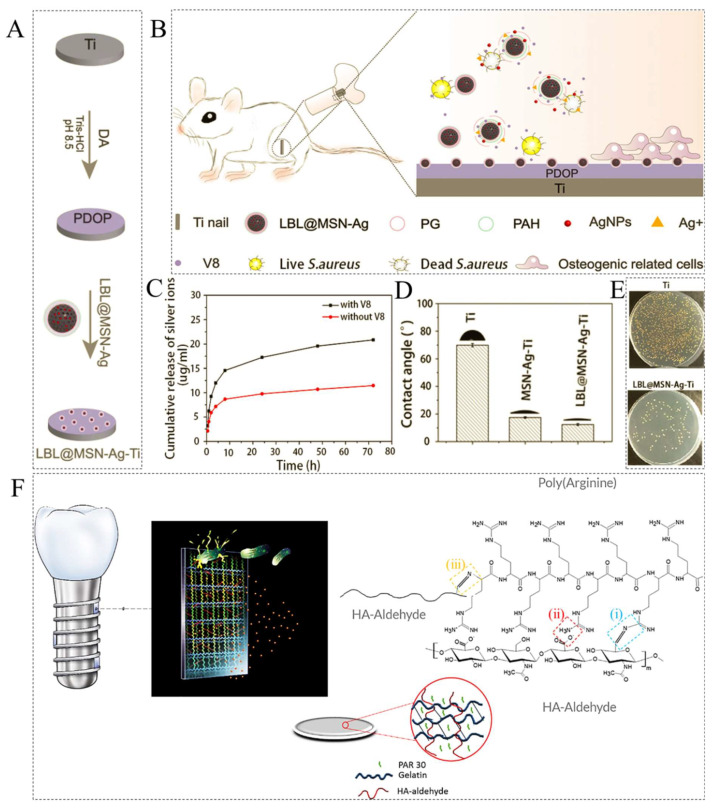
(**A**) The schematic illustration of the synthesis of LBL@MSN-Ag layer on PDOP-coated Ti substrates; (**B**) the schematic presentation of the antibacterial and osteogenic responses of the LBL@MSN-Ag layer on Ti nails in the presence of the V8 enzyme; (**C**) release profile of Ag ions from LBL@MSN-Ag nanoparticles in PBS solution with and without V8 enzyme; (**D**) water contact angles on different Ti surfaces; (**E**) spread plate images of *S. aureus* bacterium for Ti and LBL@MSN-Ag samples after implantation for one week. Reprinted with permission from [97]. (**F**) The schematic illustration of the possible interactions between PAR and HA-Aldehyde that can be coated on the dental implants to improve the angiogenesis responses and prevent peri-implantitis; (**F(i)**) the formation of an imine bond between the aldehyde group on HA and guanidine group on the PAR backbone; (**F(ii)**) ionic interactions between the carboxylic group (anions) on HA and the protonated guanidinium groups (cations) on the PAR chains; (**F(iii)**) the formation of imine bond between the aldehyde group on HA and primary amine on the PAR N-terminal side. Reprinted with permission from [102].

As another side of implant-associated infections, the bacteria present in the biofilm produce enzymes such as metalloproteinases, lipases, hyaluronidases (HAase), β-glucuronidase (β-GUS), chymotrypsin (CMS), and glutamyl endonuclease (V8) by metabolic activity on the implant surface [95,96]. These produced enzymes can be used as biological stimuli for sustained drug and antimicrobial molecules release in EZRHs [95]. Recently, Ding et al. developed EZRHs as a drug delivery platform for treating *Staphylococcus aureus* (*S. aureus*)-associated infections and accelerating bone tissue growth on a Ti implant in vivo [97]. As seen in Figure 9A, Ti substrates were modified by polydopamine (PDOP). Then MSNs loaded with Ag nanoparticles (NPs) were synthesized. In next step, MSNs-Ag NPs were capped with cationic polyallylamine hydrochloride (PAH) and biodegradable anionic poly(L-glutamic acid) (PG) films layer by layer (LBL) and named LBL@MSN-Ag. Finally, LBL@MSN-Ag spherical particles were deposited on PDOP-coated Ti. The PG is a polyamide, formed by amide linkage, and can respond to the V8 enzyme secreted by *S. aureus* during its metabolic activity [98,99]. Therefore, it can be degraded in the microenvironment of bacterial infection with a high concentration of V8 and release of Ag NPs and ions, resulting in the on-demand release of drugs along with a good antimicrobial performance at the implantation site. Moreover, the PG as a synthetic EZRH is biocompatible and has the potential to promote cell and tissue growth (Figure 9B) [100]. The cumulative release profile in Figure 9C proved that the release of Ag ions was very fast during the first 6 h of incubation in the presence of the V8 enzyme. The wettability studies (Figure 9D) showed that the LBL@MSN-Ag coating on the Ti was hydrophilic with a contact angle of 12.4°, which resulted in higher cell adhesion on the implant’s surface [101]. As shown in Figure 9E, the number of viable bacterial colonies on the LBL@MSN-Ag treated Ti were significantly lower than that of the untreated Ti implant, showing the high antibacterial ability of the EZRH coatings in vivo.

In order to reduce the implant-associated infections, Knopf-Marques et al. synthesized a smart antimicrobial coating by using the layer-by-layer method with poly(arginine) (PAR) as polycation and hyaluronic acid (HA) as polyanion to prevent bacterial colonization on Ti dental implants (Figure 9F) [102]. They found that incorporating 1.5 nmol of PAR into hydrogel coatings significantly reduced bacterial growth up to 94% with insignificant cytotoxicity. Furthermore, a human vascular endothelial cell line was stimulated to secrete vascular endothelial growth factor A (VEGFA) and to form cell–cell contacts by Gel/HA charged with PAR. The synthesized composite hydrogel can be a versatile tool for developing bacteria-responsive hydrogels with antimicrobial and therapeutic activities on the surfaces of the medical implants. In another study, PAR-decorated polydopamine nanoparticles were incorporated into the gelatin hydrogels matrix for bone tissue engineering applications [103]. The results showed that the dispersed nanoparticles not only enhanced the antibacterial and mechanical properties of the hydrogel but also provided high stability and biocompatibility for the matrix [104].

## 7. Conclusions, Challenges, and Future Directions

The introduction of stimulus-responsive effects can enhance the functionality and increase the range of applications of hydrogels in biomedical engineering. Smart hydrogels as an emerging class of material responding to external triggers, such as pH, temperature, electrical and magnetic fields, light, and concentration of biomolecules, can release the drug cargo at specific locations with controllable kinetics. Based on the last five years’ studies, the main future direction is to improve the properties of the currently developed smart hydrogels and provide them with novel, sophisticated features. In the near future, research will move toward the synthesis of programmable smart hydrogels that are capable of responding to the complex multi-stimulus and integrating multiple therapies. They are likely to have an exciting future, while some challenges are facing this field. Up to now, various smart hydrogels have been developed and introduced, but the commercialization of smart hydrogels as drug delivery systems is not convincing; only a few cases have entered into clinical use, including Jelmyto^®^ (UGN-101) as a TRH that was lunched by UroGen Pharma and received Food and Drug Administration (FDA) approval in April 2020 [105]. Indeed, given the recent advances in the pharmaceutical industry, there are still no clear legal regulations and standards for the use of smart drug-loaded hydrogels in therapeutic activities. Moreover, much progress needs to be made concerning in vivo release by modeling the release profiles before commercializing them. Finally, the field of smart hydrogels is in its infancy and its importance in designing efficient delivery systems has become clear to everyone and can create many opportunities for 21st century medicine. The next generations of smart hydrogels will most likely focus on gene-loaded hydrogels with integrated sensors to treat genetic abnormalities. Another possible research area will be pathogen-responsive hydrogels for local infection treatments. Recently, the “Gels4Bac” project has been funded by the European Research Council (ERC) and will focus on the selectively and local release of antimicrobial vesicles in the presence of specific pathogen stimuli [106].

## Figures and Tables

**Table 1:** Stimuli-responsive hydrogels with their key features, properties, and applications.

Type of Hydrogels	Examples	Key Features	Properties	Applications	References
pH-responsive	Chitosan, guar gum succinate, kappa-carrageenan, PEI, PAM, PAA, PDEAEMA, PDMAEMA, PEAAc, pHEMA, PMAA, PPAA, and PVA	pH variation results in swelling/deswelling behavior due to the changes in hydrophobicity of the polymeric chains and increase in electrostatic repulsion between chains	Biocompatibility, sustained release of incorporated drugs, increased hydrophilicity, and swelling, strong electrostatic interactions, and stability	Drug delivery, Sensing, inflammation responsive hydrogels, wound and skin healing.	[19,20]
Temperature responsive	Poloxamer, Pluronic, PAA, PNIPA, PNVCL grafted with PEO, TMC crosslinked with PEG, glycerophosphate, and methoxy poly(ethylene glycol)-poly(pyrrolidone-co-lactide)	Temperature variation disturbs the equilibrium exists between hydrophobic and hydrophilic segments of the polymeric chain and increase the sol-gel transformation rate	Unique physical properties similar to the extracellular matrix, easy functionalization with drug molecules, controlled degradation	Drug delivery, intraocular lenses, tissue engineering.	[21,22,23]
Electric field responsive	PPy nanoparticles loaded in PLGA, PEG hydrogels, Agarose, calcium alginate, carbomer, chondroitin sulphate, hyaluronic acid, partially hydrolyzed PAM, PDMA, and xanthan gum	Upon the application of an electric field, deswelling or bending takes place, based on the shape and position of the gel relative to the electrodes.	Biocompatibility, minimal invasiveness, controlled release of the cargo depending on the strength or the duration of applied electric field	Drug delivery, creams and suspensions as emulsion stabilizer, in cosmetics as thickener and stabilizer, buccal delivery.	[24,25,26]
Magnetic field responsive	Alginate-xanthan cross-linked with Ca^2+^ magnetic nanoparticles, Hemicellulose crosslinked with GGM, hemicellulose hydrogels with magnetic iron oxide (Fe_3_O_4_), methacrylate chondroitin sulfate with magnetic nanoparticles, PNIPA, and xanthan-bovine serum albumin-magnetic nanoparticles	Application of heating, mechanical deformation, or external magnetic field to magnetic nanoparticles, such as nanoparticles of magnetite, maghemite, and ferrite	Swelling behavior responsive to temperature too, some of them dispose of anisotropic properties, successful absorption and controlled release of drugs	Drug delivery, sensing, microfluidics, tissue engineering.	[27,28,29]
Light responsive	Poly [2-((4,5-dimethoxy-2-nitrobenzyl) oxy)-N-(2-(methacryloyloxy)ethyl)-N,N-dimethyl-2-oxoethan-1-aminium, HPMC, Carbopol hydrogels containing diclofenac-sodium chitosan microspheres, Azo benzene-pHEMA, azo benzene-bovine albumin, triphenylmethane leuco derivatives, and trisodium salt of copper chlorophyllin-PNIPAM^23^	External stimulus of either visible or UV light initiates sol-gel transformation	Control release, reversible and irreversible, spatiotemporal control over functional groups, reasonable strengthens according to application.	Drug delivery, optical delivery, microfluidics, self-sterilization and self-cleaning.	[30,31,32,33]
Biomolecules responsive	Insulin, phenylborate derivative 4-(1,6-dioxo-2,5-diaza-7-oxamyl) phenylboronic acid in combination with PNIPA, and poly(2-hydroxyethyl methacrylate-co-N,N-dimethylaminoethyl methacrylate) in combination with glucose oxidase	Changes in biomolecule concentration and pH in hydrogel as a self-regulated, can expand the polyelectrolytes resulting in swelling/deswelling behavior.	Enzyme responsive, achieves molecular recognition, high affinity, and specificity, controlled release, biocompatibility.	Drug delivery, insulin-delivery system, cell culture, sensing, tissue engineering.	[34,35,36]

Abbreviations (pH-responsive hydrogels): Poly(ethyleneimine) (PEI); Poly(acrylamide) (PAM); Poly(acrylicacid) (PAA); Poly(diethylaminoethyl methacrylate) (PDEAEMA); Poly(dimethylaminoethyl methacrylate) (PDMAEMA); Poly(ethylacrylic acid) (PEAAc); Poly(hydroxyethyl methacrylate) (pHEMA); Poly(methacrylic acid) (PMAA); Poly(propylacrylic acid) (PPAA); Poly(vinyl alcohol) (PVA). Abbreviations (temperature-responsive hydrogels): Poly(N-isopropylacrylamide) (PNIPA); Poly(N-vinyl caprolactam) (PNVCL); Poly(ethylene oxide) (PEO); N-trimethyl chitosan chloride (TMC); Poly(ethylene glycol) (PEG). Abbreviations (electric field-responsive hydrogels): Polypyrrole (PPy); Poly lactic-co-glycolic acid (PLGA); Polydimethylaminopropyl acrylamide (PDMA). Abbreviations (magnetic field-responsive hydrogels): O-acetyl-galactoglucomannan (GGM); Poly(N-isopropylacrylamide) (PNIPA). Abbreviations (light field-responsive hydrogels): Hydroxypropyl methylcellulose (HPMC); Poly(N-isopropylacrylamide) (PNIPAM).

## Data Availability

Not applicable.

## Abbreviations

PRHs	pH-responsive hydrogels
MNs	Stainless steel microneedles
ROS	Reactive oxygen species
DEX	Dexamethasone
PEG	Poly(ethylene glycol)
SEM	Scanning electron microscopy
Ti	Titanium
IB	Ibuprofen
MSNs	Mesoporous silica nanoparticles
TRHs	Temperature-responsive hydrogels
LCST	Lower critical solution temperature
UCST	Upper critical solution temperature
PDEAM	Poly(N,N-diethyl acrylamide)
PNIPAM	Poly (N-isopropylacrylamide)
PMVE	Poly(methylvinylether)
PVC	Poly(N-vinylcaprolactam)
β-CD	β-cyclodextrin
Ad-PEG	Adamantyl-terminated poly(ethylene glycol)
α-CD	α-cyclodextrin
DOX	Doxorubicin
HMPC	Hydroxypropyl methylcellulose
CS	Chitosan
Gly	Glycerin
CGHH	Hydroxypropyl methylcellulose/Chitosan/Glycerin composite
NT	Nanotube
BMP	Bone morphogenetic protein
G′	Storage modulus
G″	Loss modulus
ERHs	Electrically responsive hydrogels
CP	Chitosan-graft-polyaniline
OD	Oxidized dextran
MRHs	Magnetically responsive hydrogels
SPIONs	Superparamagnetic iron oxide nanoparticles
LRHs	Light-responsive hydrogels
NIR	Near-infrared radiation
BPNSs	Black phosphorus nanosheets
GRHs	Glucose-responsive hydrogels
IVDD	Intervertebral disc degeneration
HA	Hyaluronic acid
Met	Metformin
ERHs	Enzyme-responsive hydrogels
HAase	Hyaluronidases
β-GUS	β-glucuronidase
CMS	Chymotrypsin
V8	Glutamyl endonuclease
PDOP	Polydopamine
Ag NPs	Silver nanoparticles
PAH	Polyallylamine hydrochloride
PG	Poly(L-glutamic acid)
LBL	Layer by layer
PAR	Poly(arginine)
VEGFA	Vascular endothelial growth factor A
FDA	Food and Drug Administration
ERC	European Research Council
